# Smoking cessation behavioural therapy in disadvantaged neighbourhoods: an explorative analysis of recruitment channels

**DOI:** 10.1186/s13011-015-0024-3

**Published:** 2015-07-31

**Authors:** Fiona E. Benson, Vera Nierkens, Marc C. Willemsen, Karien Stronks

**Affiliations:** Department of Public Health, Academic Medical Centre, University of Amsterdam, Meibergdreef 9, 1105 AZ, Amsterdam, The Netherlands; Department of Public Health and Primary Care, LUMC, Hippocratespad 21, 2333 RC, Leiden, The Netherlands; Department of Health Promotion, CAPHRI School for Public Health and Primary Care, Maastricht University, Minderbroedersberg 4-6, 6211 LK, Maastricht, The Netherlands

**Keywords:** Socioeconomic factors, Neighbourhood, Reach, Referral, Smoking cessation, Attendance

## Abstract

**Background:**

The optimum channel(s) used to recruit smokers living in disadvantaged neighbourhoods for smoking cessation behavioural therapy (SCBT) is unknown. This paper examines the channels through which smokers participating in a free, multi-session SCBT programme heard about and were referred to this service in a disadvantaged neighbourhood, and compares participants’ characteristics and attendance between channels.

**Methods:**

109 participants, recruited from free SCBT courses in disadvantaged areas of two cities in the Netherlands, underwent repeated surveys. Participants were asked how they heard about the SCBT and who referred them. Participant characteristics were compared between five channels, including the General Practitioner (GP), a community organisation, word of mouth, another health professional, and media or self-referred. Whether the channels through which people heard about or were referred to the service predicted attendance of ≥4 sessions was investigated with logistic regression analysis.

**Results:**

Over a quarter of the participants had no or primary education only, and more than half belonged to ethnic minority populations. Most participants heard through a single channel. More participants heard about (49 %) and were referred to (60 %) the SCBT by the (GP) than by any other channel. Factors influencing quit success, including psychosocial factors and nicotine dependence, did not differ significantly between channel through which participants heard about the SCBT. No channel significantly predicted attendance.

**Conclusion:**

The GP was the single most important source to both hear about and be referred to smoking cessation behavioural therapy in a disadvantaged neighbourhood. A majority of participants of low socioeconomic or ethnic minority status heard about the programme through this channel. Neither the channel through which participants heard about or were referred to the therapy influenced attendance. As such, concentrating on the channel which makes use of the existing infrastructure and which is highest yielding, the GP, would be an appropriate strategy if recruitment resources were scarce.

## Introduction

Smoking is the main modifiable behavioural risk factor for the global burden of non-communicable disease [1]. Compared with people living in advantaged areas, people living in disadvantaged areas in high-income countries, smoke more [2–4] and are less likely to quit [5]. Proven effective interventions exist, such as multi-session smoking cessation behavioural therapy (SCBT) with or without pharmacotherapy [6], however, in order to benefit from these, smokers in disadvantaged areas must first be reached and recruited [7].

Targeting reach and recruitment activities to disadvantaged areas has been shown to be successful in recruiting smokers in the UK [8]. Apart from this, however, there is currently scant evidence on how smokers in such areas are best reached and recruited [9]. A recent Cochrane review highlighted the areas within this field that need more attention, which included identifying those recruitment strategies (or different combinations of particular recruitment strategies) that work better for different population groups [10].

In recruitment strategies, a distinction needs to be made between the channel through which participants had their attention captured (or heard about) an intervention [11], and the channel through which they were referred to the intervention. These two steps can happen through the same channel (e.g. hearing about and being referred by the General Practitioner (GP)), or through different channels (e.g. hearing about it from the media and self-referring). Though the GP is used to recruit smokers in disadvantaged areas [12, 13], the literature indicates a broad range of other channels including use of other health professionals [14], use of existing community organisations [15, 16], use of media [13] and word of mouth [16].

In addition to the channels that are used by smokers in disadvantaged areas to reach smoking cessation services, the characteristics of the smokers themselves are also important to map. First, it is possible that multiple channels used in a geographically targeted area may each attract different groups of smokers, with different a priori quit success rates. We know that factors such as social support [17–20], self-efficacy [21, 22], motivation [17, 23, 20], and nicotine dependence [17, 24] can influence quit success. We also know that they can differ by individual socioeconomic status [17, 25]. Different channels may deliver participants who exhibit differences in socio-demographic characteristics and nicotine dependence [11], and possibly also in psychosocial factors such as social support, self-efficacy, and motivation. This might be the case because some strategies, from the viewpoint of the smoker, are pro-active (e.g. self-referring after reading a newspaper advertisement), and others are reactive (e.g. hearing from and then being referred by the GP) [26]. It is possible that a reactive strategy (from the viewpoint of the smoker), for example being opportunistically urged to stop by an authority figure, such as the GP during an appointment about a different matter, may deliver participants who feel externally controlled and thus have lower levels of self-determination, which leads to lower levels of motivation and self-efficacy [27]. In contrast, a proactive strategy (from the viewpoint of the smoker), such as responding to a media message about a smoking cessation programme may deliver participants who have internalized this goal and are acting toward it, exhibiting more self-determined behaviour, and possibly higher motivation and self-efficacy [27].

Second, once a participant is successfully recruited, attending more sessions of SCBT predicts quit success [28]. It is possible that recruitment channel affects attendance through socio-demographic characteristics of those recruited through the channel [29, 30, 17] or through the various levels of extrinsic motivation which are possibly called into action by recruitment channel. More detailed knowledge of which participants are recruited through possible channels and how these channels influence attendance would therefore allow interventionists to be confident that they are spending their money in an effective way and might contribute to a more cost-effective intervention.

Therefore, within the context of those who are successfully recruited for smoking cessation behavioural therapy (SCBT) in disadvantaged areas, this study will explore the channels through which participants heard about the SCBT and were referred to this service, and whether these channels recruited a different group of smokers. This study uses data from an intervention carried out in disadvantaged neighbourhoods in the Netherlands. Free behavioural therapy with optional reimbursed pharmacotherapy was offered. In these geographically defined areas, campaigns were launched aiming to recruit smokers living in these areas. These included use of the GP, other health professionals, community organisations, media and word of mouth. We will consider which channels were used, the characteristics of the smokers using these channels and whether channel predicted attendance of the smoking cessation service. A descriptive approach has been used in this article due to its explorative nature, as there is little evidence that any single channel is either the most effective or the most favoured by people from lower socioeconomic status (SES) groups.

## Methods

### Participants

The 109 participants in this study were recruited among participants in SCBT as implemented in two large cities in the Netherlands, here referred to as Cities A and B. There were two sites in each city. Three sites were amongst the 40 most disadvantaged areas in the Netherlands according to a classification of the Dutch government [31], two of which had large ethnic minority populations. City B, also included an area which scored in the lowest 10 % of areas in the Netherlands when compared on education, income and job prospects of participants [32]. Recruitment of study participants took place from May 2011–October 2013.

### Channels

In each city, a broad range of activities were implemented to recruit participants in the SCBT through different channels, including GP, other health care professionals, and media. In one city (City A) community organisations were also approached and participants were also encouraged to recruit their own contacts.

Local GP’s actively told their patients about, and referred them to, the SCBT. In both cities, a staff member from the SCBT provider liaised with local GPs to ensure that practices in the areas knew about the programme and would actively refer patients. In City A, this liaising staff member also held a feedback session for the GPs during the study period and sent out letters reminding GPs about the services later in the study period. Twenty-one different GPs from 13 different practices referred patients to the SCBT.

Other health professionals (HP) such as practice support nurses and pharmacists actively told patients/clients about the SCBT and referred them. In the GP practices involved, practice nurses were informed about the programme by SCBT provider staff members or their practice managers, and were able to refer patients to the programme. Information was provided in both cities by local pharmacists (one in each area) and community nurses (10 in total).

Community organisations (e.g. religious/cultural/ sporting/food bank) were targeted by the SCBT Coach or Project Leader to gain the support of the leadership or to directly approach participants. Twenty-eight community organisations (with a combined following of at least 3700 people) were approached leading to 90 contact moments and 36 site visits, some of which involved making presentations to the members, manning information stalls at the community organisation’s own events, or discussions with the key leaders.

Media sources were also used. Posters and flyers were available in various locations in each area including GP surgery, pharmacy, community centre and library. In City A these were also distributed at many of the community organisations mentioned above. These were available 2–3 months prior to each course (City B) or shortly before the start of the first course (City A) and remained visible for the entire recruitment period. These were in Dutch (both cities) and Turkish (City A). The local newspaper carried either a story about the programme (City A) or an advertisement of the programme (City B) during the recruitment period. Information about the course was available on a local (specific to the local SCBT provider) or national (smoking cessation organisation (STIVORO)) website. In City A only, information about the SCBT and how to sign up was displayed on a television screen in the waiting room of a general practice where the SCBT was run.

Recruitment of participants through word of mouth was actively promoted in City A, where participants of the SCBT were encouraged to recruit other participants.

### SCBT intervention

The SCBT was offered by telephone (7 sessions) or in a group setting (9 sessions, +/- two additional sessions on participant-chosen lifestyle topics). A date on which participants would quit was agreed with their counsellor. City B offered group counselling only. All participants in group counselling were offered pharmacotherapy, as were those smoking >10 cigarettes per day in telephone counselling. For part of the study period (in 2011 and 2013) pharmacotherapy could be obtained free of charge, as long as there had been contact with the GP.

### Data collection

All participants >18 years of age who signed up for SCBT were asked if they would participate in the research. All those who expressed interest in participating were contacted. Participants were contacted by telephone, a minimum of 4 times. If participants were unable to be contacted, or refused to take part in the research, they were categorised as ‘non-response’. Of the 270 participants who signed up for SCBT, 161 agreed to be contacted. Of those, 49 were non-response and three were excluded (due to hospitalisation, only participating to support a friend and not actually wishing to quit smoking, and because the coach did not feel that the participant was ready to quit and thus did not enrol him in the course, respectively), leaving 109 participants. Of these, 27 dropped out prior to the second questionnaire, however they were included in all of the analysis except that of attendance, which required information from the second questionnaire.

Participants in the research were invited to undertake 4 questionnaire-guided face-to-face interviews at the following intervals: prior to SCBT (baseline), and then 4–6 weeks, 6 months and 1 year after their agreed quit date. They were given a €10 honorarium per interview. Interviews took place in Dutch or Turkish, according to participant preference. Data for all variables except attendance (second questionnaire) was collected in the baseline questionnaire. Informed consent was obtained from all participants. Medical ethical approval for this study was not required under Dutch law as no intervention was undertaken by the researchers.

### Measures

Socio-demographic variables were measured as follows: gender (male/female), age (age at start of course), relationship status (dichotomised: ‘partner’ (married/registered partnership, living together)/‘No partner’ (unmarried/never married, divorced or widower)), ethnicity (as per definition of Statistics Netherlands [33]: Dutch (born in the Netherlands with two parents born in the Netherlands)/non-Dutch (first generation (born outside the Netherlands with one or both parents born outside the Netherlands) or second generation (born in the Netherlands with one or both parents born outside the Netherlands)). Educational level was used as a proxy for SES, and was measured in tertiles (none or primary/secondary/tertiary).

Participants were asked: ‘How did you hear about the stop smoking programme?’ For each of the channels used, participants had to indicate whether they had heard through this channel or not.

Participants were also asked: ‘Who referred you to the stop smoking programme?’ Possible answers were: GP, member of a community organisation, or someone else. They were further asked about the name of the individual/organisation and, in the case of an answer of ‘someone else’, what their relationship was with the person.

Self-efficacy expectations were measured with 6 questions, such as: ‘You feel stressed or tense. Will you succeed in not smoking?’ These questions were developed specifically for ethnic minority groups in The Netherlands and have been validated [34, 35]. Answers were on a five point Likert-type scale from ‘definitely’ to ‘definitely not’ (scored 1–5 respectively), with an additional answer of ‘don’t know’ (scored 0). Cronbach’s α was 0.85. The possible range after summation was 6–30.

Social support expectations of partner, children, extended family and friends respectively (4 items) were measured with questions such as: ‘Will you be supported by your partner if you stop smoking?’ Questions on perceived social support, also from the same source as those of self-efficacy expectations (above), were modified by researchers to clarify how much social support people expected to receive, as this may have an impact on attendance [36] and smoking cessation behaviour [18, 19]. Possible answers were ‘a lot’ , ‘average’ , ‘little’ , ‘no’ (scored 3 – 0 respectively) or ‘not applicable’ (scored 0). Cronbach’s α was 0.46. The possible range after summation was 0–12.

Motivation to quit was measured with the question: ‘How motivated are you to stop permanently during this programme?’ This question was chosen by researchers. Answers were on a scale from 1–10, where ‘1’ was ‘not motivated’ and ‘10’ was ‘highly motivated’.

Nicotine dependence was measured with the Fagerstrøm Test for Nicotine Dependence with revised scoring [37]. The answers to the 6 questions were summed (possible range 0–10) and then participants were categorised according to score from ‘low dependence’ (0–4), moderate dependence (5) and ‘high dependence’ (6–10).

Attendance was measured with the question: ‘How many group sessions have you attended?’ or ‘How many telephone conversations have you received?’ This was asked in the second questionnaire which was done 4–6 weeks after participants agreed quit date, thus at a time when participants attending fully would have completed a minimum of 5 telephone counselling sessions, or a minimum of 7 of group counselling sessions. For this reason, attendance was dichotomised at ≤3 and ≥4 sessions, according to the Dutch clinical guidelines [28]. At the time of the second questionnaire, participants falling into the first group would have had to have missed at least 2 sessions for telephone and at least 4 sessions for group counselling.

### Analysis

Analysis was done using IBM SPSS Statistics Version 21 (Release 21.0.0.1). To analyse which channels were used, we first sorted participants answers about the channel through which they heard about the SCBT and were referred to the SCBT into meaningful categories. We then determined how many participants heard and were referred through each channel.

To explore the characteristics of participants, characteristics were considered per channel, including socio-demographic characteristics and psychosocial variables influencing quit success. To test whether socio-demographic characteristics differed per channel (some of which contained small numbers), the extended Fisher’s Exact Test was applied. Participants who had heard from >1 source were excluded for this testing. For self-efficacy expectations, answers to all of the six questions making up this variable were summed per individual. After this, the mean and 95 % Confidence Interval per reach and referral channel were determined. This method was also used for social support expectations. The mean and 95 % Confidence Interval was determined for motivation to quit and nicotine dependence, per reach and referral channel. Differences were considered to be significant if confidence intervals did not overlap.

To determine whether the channel predicted attendance of ≥4 sessions of SCBT, logistic regression analysis was used. Each of the multivariate models was corrected for age, gender, ethnicity, educational level and counselling type. A *p*-value of <0.05 was considered significant.

## Results

The characteristics of the 109 participants can be found in Table 1. The average age of participants was 48 years. Approximately half were female and over half were from ethnic minority groups. Just over a quarter of the participants had no or primary education only.

**Table 1:** Participant characteristics

		All participants (*N* = 109)
*n*(%)		
Gender	Male	57(52)
	Female	52(48)
Relationship status	Partner	57(52)
	No partner	52(48)
Ethnicity	Dutch	45(41)
	Non-Dutch	64(59)
Educational level	Low	32(29)
	Medium	61(56)
	High	15(14)
Mean(SD)		
Age		48 (13.2)

**Fig. 1:**
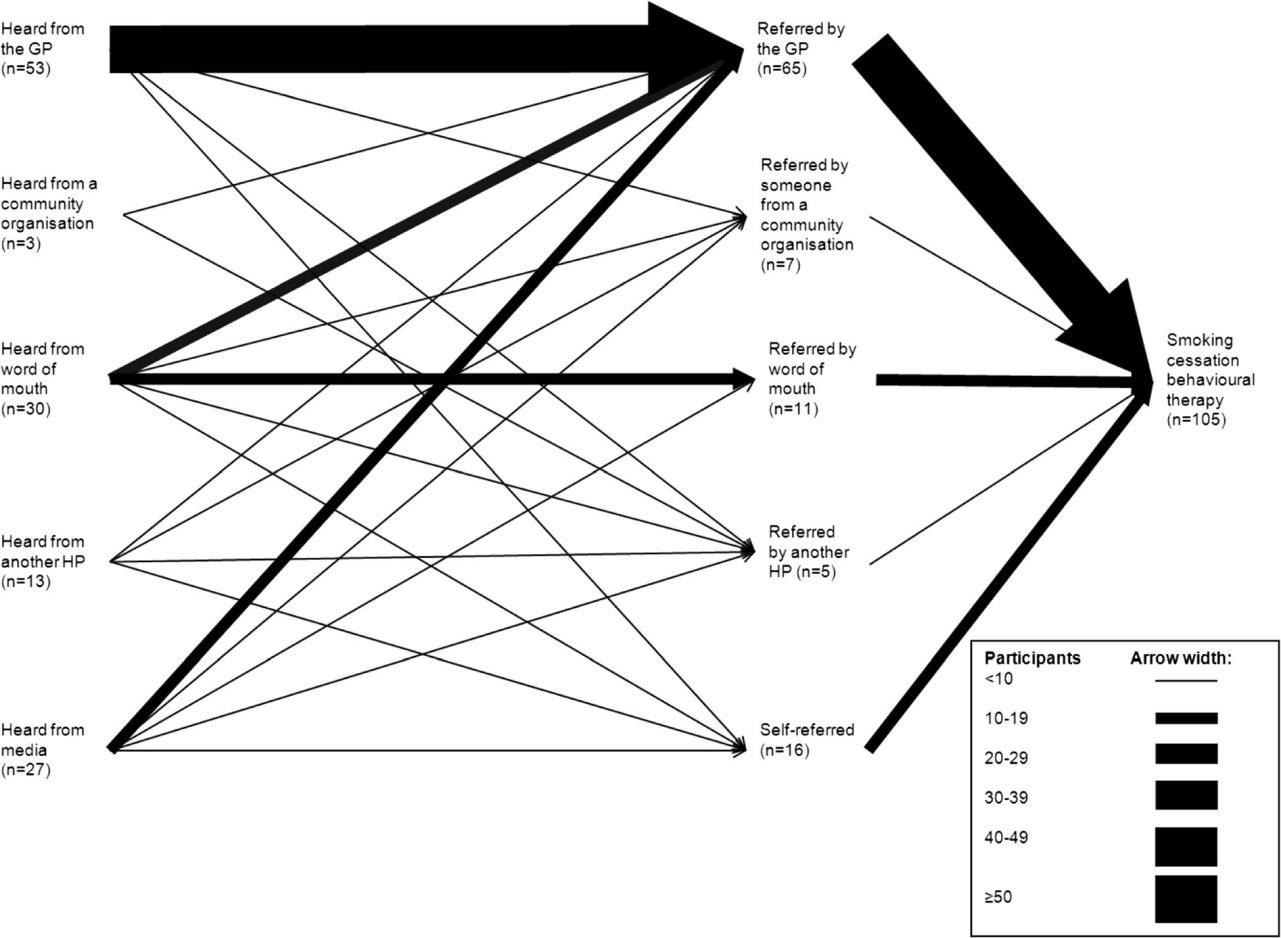
Channels through which participants heard about and were referred to the SCBT

**Fig. 2:**
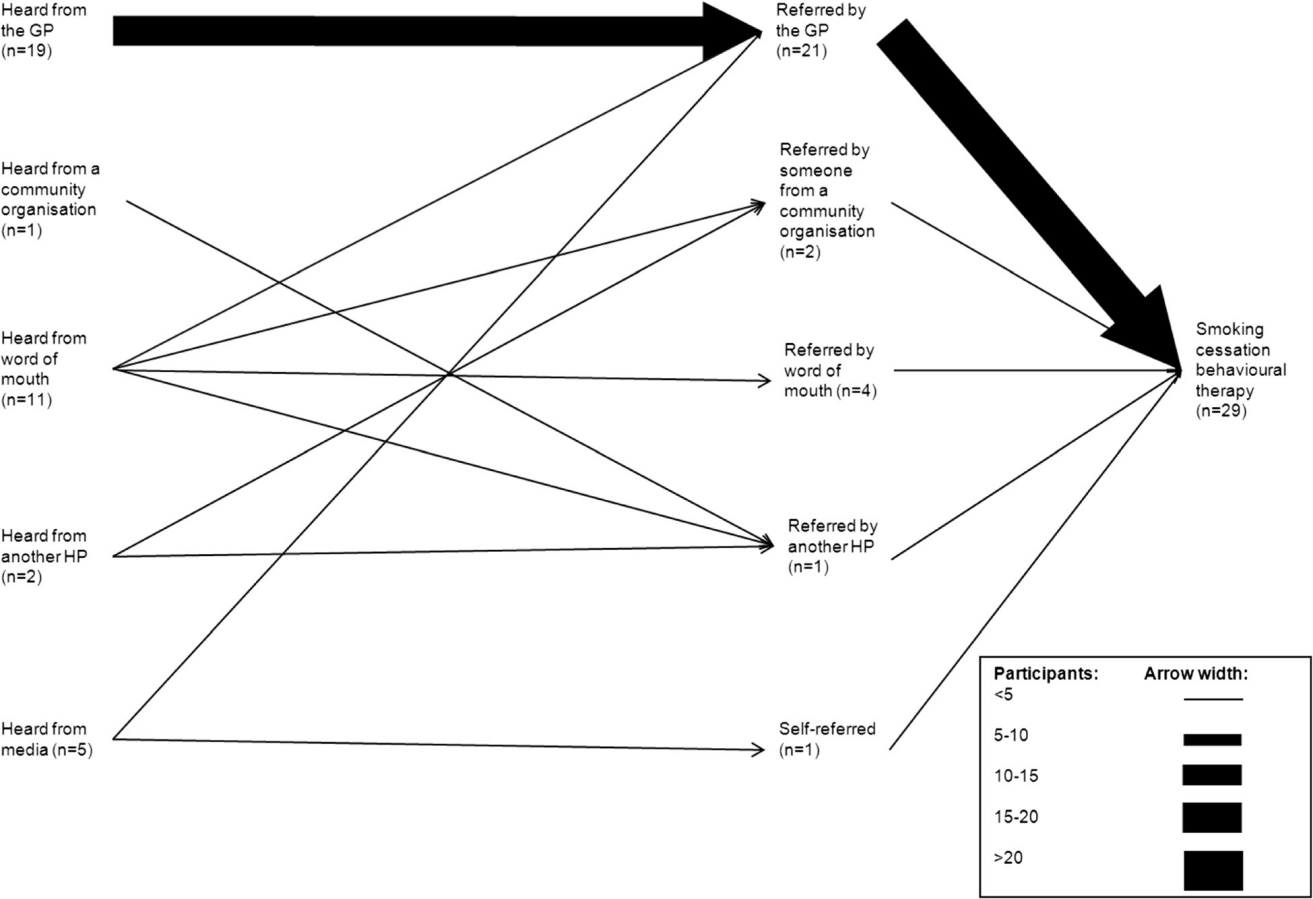
Channels through which participants with no/primary education heard about and were referred to the SCBT

The channels through which participants heard about and were referred to SCBT can be viewed in Fig. 1. The most common way to hear about the SCBT was through the GP (49 %), The majority of participants heard about the programme through a single source (82 %), which was most often the GP (39 %), with a minority hearing from 2 (15 %) or 3 (3 %) sources. Of those who heard from >1 source, the majority heard from the GP and another source (56 %), which was most often a media source (39 %). When we restricted the analyses to those with no/primary education only, we found the same pattern (Fig. 2). As only a small number of participants heard from a community organisation, this category has been removed from further analysis.

**Fig. 3:**
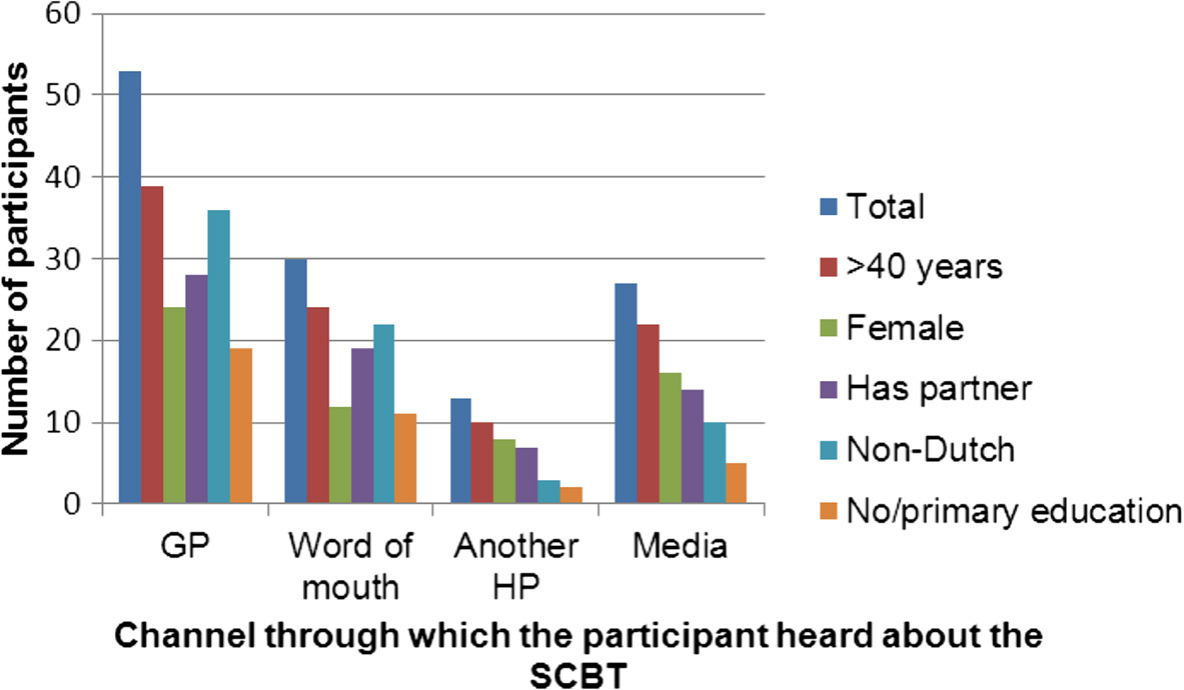
Socio-demographic characteristics of participants who heard about the SCTB through a specific channel

**Fig. 4:**
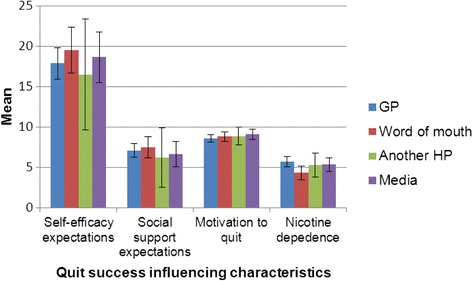
Comparison of the means (and confidence intervals) of variables influencing quit success per channel through which participants heard about the SCTB

We observed differences in the characteristics of participants that heard about the SCBT through a particular channel (Fig. 3). The percentage of participants in each channel significantly differed by ethnic background (Fisher’s Exact Test = 19.19, *p*-value = 0.00, two-sided, 4x2 table) and educational level (Fisher’s Exact Test = 12.22, *p*-value = 0.04, two-sided, 4x3 table). A greater proportion of participants of low educational level and ethnic minority background heard about the SCBT through the GP (36 % and 68 % respectively) and word of mouth (37 % and 73 % respectively), than was the case in those hearing through another HP (15 % and 23 % respectively) and media (19 % and 37 % respectively) sources. There were no significant differences in the motivation, self-efficacy or social support expectations or nicotine dependence of those who had heard through different channels, as determined by comparing the means and 95 % Confidence Intervals (Fig. 4).

Reach and recruitment channels did not appear to predict frequency of attendance of multiple sessions of the SCBT (≥4 sessions) (Table 2), where *p*-values were calculated using the Wald chi-square test. Controlling for possible correlates did not change estimations (results not shown).

**Table 2:** Univariate logistic regression models testing the predictive value of channel on attendance of ≥4 sessions

		Channel through which participants heard about the SCBT OR(95 % CI)	*df*	*p-*value*	Referral channel OR(95 % CI)	*df*	*p*-value*
GP	No	1.0	1		1.0	1	
	Yes	0.92(0.33–2.58)		0.88	0.56(0.20–1.57)		0.27
Word of mouth	No	1.0	1		1.0	1	
	Yes	1.23(0.38–3.98)		0.73	1.25(0.23–6.94)		0.80
Another HP	No	1.0	1				
	Yes	1.53(0.29–8.17)		0.62			
Media	No	1.0	2				
	Yes	0.93(0.29–3.0)		0.91			
	Missing	0.45(0.14–1.44)		0.18			
Self-referred	No				1.0	1	
	Yes				2.02(0.51–8.00)		0.32

*OR* Odds Ratio, *CI* Confidence Interval, *df* = degrees of freedom

*p-values are based on the Wald chi-square test, degrees of freedom = 1 or 2

**p-value <0.05 is considered significant

## Discussion

We have found that the GP was the main way through which participants both ‘heard about’ and were ‘referred’ to SCBT courses in a disadvantaged areas. More participants of low educational level as compared to those of high educational level heard through the GP and word of mouth. Also, the majority of participants were referred through the GP. We did not find a significant difference in motivation, self-efficacy or social support expectations and nicotine dependence in those who ‘heard about’ the SCBT through different channels. Finally, the channel through which people heard about or were referred to the SCBT did not predict attendance.

A major strength of this study is that it was done in a usual-care setting of an SCBT intervention offered within the context of disadvantaged areas, rather than in a randomised controlled trial setting where participants are often screened by strict selection criteria and treatment is strictly monitored [38]. As such, we are confident that the responses truly indicate the various pathways through which a range of participants in the community end up at the SCBT.

Another strength of this study is that despite the two cities differing considerably in the proportion of participants from ethnic minority backgrounds, the conclusion, that the GP was the main way through which participants heard about or were referred to the SCBT, is true in both cities (data not shown here). This may make the conclusion generalisable to different types of disadvantaged areas.

Some limitations must, however, be taken into consideration when considering the results of this study. Firstly, due to changes in the Dutch basic health insurance package, during part of the study pharmacotherapy was reimbursed with a GP referral (2011 and 2013) and during the other year (2012), it was not reimbursed. This may have led to participants going to the GP for referral during the reimbursement period after hearing about the SCBT elsewhere, thus increasing the number of participants being referred through the GP. However, we would not expect this to affect the way people heard about the course in the first place.

We missed data for some questions. Due to participant drop-out (27) or missing answers (3), 30 participants (28 %) lacked attendance data. Compared to the total participants (Table 1), those who lacked attendance data were more often male, married, and non-Dutch (Appendix 1), however they were reflective of the total group from City A (Appendix 2). We do not think the missing data biased our results because each group for which data were missing in one of the measurements, were similar to the other participants from their city (Appendix 1, & Appendix 2).

The Cronbach’s alpha for social support expectations was low and thus possibly not reliable. However, the results showing lack of difference in social support expectations also mirror the relationships for other quit success influencing factors.

It is always possible that certain reach or referral channels were not exploited to their full potential, leading to low gain from that channel and thus biasing the results. For example, it is possible that some of the community organisations were visited too far in advance of the SCBT availability. We do not think that this biased the results, however, as the efforts in each of the channels were of a similar intensity, or, if of a lesser intensity (i.e. word of mouth), probably reached a similar number of motivated members of the target group.

Attendance was measured toward, but prior to, the end of the course. It is possible that some participants who had missed several sessions at the start then attended well at the end of the SCBT and were incorrectly categorised into the <4 sessions category while they actually attended more sessions. We would not expect this to lead to bias, however, because we would not expect any misclassification to be greater in any particular channel.

Several implications for recruitment activities in disadvantaged areas arise from this research. Firstly, while a considerable proportion of the individual SES of the residents of a disadvantaged area may be low, this will not be the case for all residents [12]. Just over a quarter of our participants were of low educational level. In the UK, where geographical targeting is also used, a 10 year review of the national stop smoking services found that 54 % of smokers treated belonged to disadvantaged groups (measured by eligibility for free prescriptions) [8]. This proportion is higher than we found in this study, though educational level of participants alone is not available in the UK study, thus we cannot directly compare this result with that of our own study. Because geographical targeting results in channels being open to all residents of the area, those with individual-level low SES make up only a proportion of those who participated. It is possible that further targeting of those with individual-level low SES characteristics (e.g. low income or low educational level) within disadvantaged areas is required to gain a larger proportion of participants of individual-level low socioeconomic status.

General practices are often used to recruit smokers in disadvantaged areas [39, 12, 13]. The question posed by a recent Cochrane review, with regard to which strategies work in low SES groups [10], can be partly answered by our study, where the majority of participants of low SES both heard about and were referred to the SCBT by their GP. The findings of this review, that personalised, proactive (from the point of view of the GP) approaches with increased contact time were most effective [10] is echoed by our highest yielding channel, the GP. However, we cannot comment directly on effectiveness because we do not know how many participants were reached by this channel overall.

One of the tasks GPs are recommended to perform in the Netherlands is giving smoking cessation advice [40]. If they find a patient is willing to quit, but do not have the time to provide smoking cessation support themselves, they are recommended to refer the patient to an SCBT programme provider or to arrange a follow-up appointment with their practice nurse, if they are properly trained in provision of intensive smoking cessation counselling [40]. However, GPs do not perform this task systematically [41]; one study which videotaped general practice appointments found the Dutch GPs in 2007–2008 discussed smoking in a minority of consultations [42]. We would recommend that GPs be encouraged to follow the guidelines, and that they be made aware and regularly reminded of the SCBT offerings available in their local area to whom they can refer.

Another strategy which has been used to reach smokers in disadvantaged communities is use of existing community organisations (e.g. sports, religious or cultural [43, 15]). This can be done by personally approaching members or by attempting to gain support from, or influence the attitudes of, the most influential members of these organisations. Despite interactions with many organisations, only 3 % of our participants indicated that they had heard about and 6 % indicated that they were referred to the SCBT through community organisations. Thus in this study, there is limited direct additional benefit from the community organisation channel.

Personal contacts can be very important in disadvantaged areas, and especially amongst those of low socioeconomic background [44], and this has also been shown in some ethnic minority groups [45, 46]. A Swiss study found that in a disadvantaged ethnic minority group the 12 month success rate was very high after recruitment using a personal contacts approach [47]. Thus it might be expected that ethnic minority participants in disadvantaged areas would hear about smoking cessation opportunities from friends and relatives. This was confirmed by our research where 28 % of participants heard through ‘word of mouth’, most of whom were non-Dutch, suggesting that this channel may be better utilized in this group.

Media is also often used to recruit smokers in disadvantaged areas [13, 43] and this can include posters, flyers, or advertising in newspapers etc. McDonald (1999), in a review, found that in the general population interpersonal approaches, such as through the GP, were more effective than media, however, more effective still, was a combination of the two [48]. Though we cannot comment on effectiveness, in our study, a quarter of participants had heard about the SCBT through the media, and most of these were then referred by the GP. A majority of them were of medium and high educational level. We also found that of those who heard from more than one source, some heard from the GP and media, however, unlike McDonald, this combination was not the most successful approach, as most people only heard from a single source.

The motivation, self-efficacy and social support expectations, and nicotine dependence of those using various channels to hear about the SCBT were similar in this study. The highest yield in both hearing about and being referred, in both the total study population and those of low educational background, comes from the GP, and there is no negative impact on attendance of using this source. There were some differences in socio-demographic characteristics (e.g. a majority of participants of ethnic minority status heard through the GP), and when this is considered in the context of most participants only hearing from a single source, some groups may be under represented if this channel is used exclusively. Thus we recommend concentrating mainly on the GP channel in disadvantaged areas, and adding further channels if financially possible.

## Conclusion

The main way that most participants heard about and were referred to smoking cessation behavioural therapy in disadvantaged areas of the Netherlands was through the GP. Psychosocial factors influencing quit success and attendance were similar between channels through which participants heard about the SCBT. Focusing on reach and recruitment through the GP would be a reasonable strategy for interventionists with limited resources. When implementing smoking cessation services good contacts with the GP should be made and maintained in disadvantaged areas, such that GPs know which treatment types are available in their local area and where they can refer their patients to.

## Appendix 1

**Table 3:** Characteristics of participants with missing data

		Participants missing attendance data (*n* = 30)
*n*(%)		
Gender	Male	19(6)
	Female	11(37)
Relationship status	Partner	22(73)
	No partner	8(27)
Ethnicity	Dutch	8(27)
	Non-Dutch	22(73)
Educational level	None or Primary	12(40)
	Secondary	15(50)
	Tertiary	3(10)
Mean(SD)		
Age		47.50(12.09)

## Appendix 2

**Table 4:** Characteristics of participants recruited from Cities A and B

		City A (*n* = 82)	City B (*n* = 27)
*n*(%)			
Gender	Male	48(59)	9(33)
	Female	34(42)	18(67)
Relationship status	Partner	45(55)	12(44)
	No partner	37(45)	15(56)
Ethnicity	Dutch	19(23)	26(96)
	Non-Dutch	63(77)	1(4)
Educational level	None or Primary	30(37)	2(7)
	Secondary	44(54)	17(63)
	Tertiary	8(10)	7(26)
Mean(SD)			
Age		46.33(11.84)	52.52(16.17)
